# Privacy-aware learning analytics for exploratory behavior through federated and explainable AI

**DOI:** 10.3389/frai.2026.1898699

**Published:** 2026-09-15

**Authors:** Rommel Gutierrez, Aracely Mera-Navarrete, Ana Cristina Villegas, Vanessa Guevara, William Villegas-Ch

**Affiliations:** 1 Escuela de Ingeniería en Ciberseguridad, Facultad de Ingeniería y Ciencias Aplicadas, Universidad de Las Américas, Quito, Ecuador; 2 Escuela de Comunicación, Facultad de Jurisprudencia, Ciencias Sociales y Humanidades Andrés F. Córdova, Universidad Internacional del Ecuador, Quito, Ecuador; 3 Unidad Educativa Dr. Emilio Uzcátegui, Área de Lengua y Literatura, Quito, Ecuador; 4 Departamento de Informática, Universidad Americana de Europa (UNADE), Cancún, Mexico

**Keywords:** behavioral modeling, educational data mining, explainable artificial intelligence, federated learning, learning analytics, privacy-preserving AI, trustworthy AI

## Abstract

Learning analytics platforms increasingly use behavioral traces to model engagement, navigation, and self-directed learning dynamics in virtual learning environments (VLEs). However, most current approaches prioritize predictive accuracy, centralize sensitive student records, and rarely analyze the stability of exploratory representations under distributed configurations. This study proposes a privacy-aware framework integrating behavioral learning analytics, federated learning (FedAvg), and Explainable AI to reconstruct informal and exploratory patterns from VLE traces while reducing direct exposure to raw data. Using OULAD as the primary dataset, the framework reconstructed 29,228 student–module–presentation units from 10,900,970 interactions and generated a behavioral proxy based on resource diversity, revisits, temporal dispersion, and non-evaluative activity. In the operational proxy-recoverability configuration, Logistic Regression achieved *F*1 = 0.977 and AUC-ROC = 1.000; this near-perfect value is interpreted as an upper-bound recovery of a transparent behavioral proxy rather than as external prediction of an independently observed construct. Importantly, the reduced scenario maintained *F*1 = 0.905 and AUC-ROC = 0.991 after excluding direct proxy components. Under distributed training, FedAvg preserved utility comparable to that of the centralized MLP (*F*1 = 0.671; AUC-ROC = 0.886), maintaining Overlap@10 = 1.000 and a Spearman correlation of ρ = 0.985 between centralized and federated explanatory rankings. The results suggest that exploratory representations can preserve analytical utility and explanatory stability in lightweight federated educational scenarios.

## 1 Introduction

Virtual learning environments (VLE) record a layer of student activity that is rarely limited to assessment submission or linear content consumption. Within a single learning trajectory, repeated access to resources, participation in forums, navigation between subpages, out-of-sequence queries, revisits, and temporally distributed activity can coexist. These signals are relevant because they describe how students explore the educational environment rather than only whether they pass or fail, particularly in scenarios associated with exploratory behavior, self-directed learning, and autonomous navigation on digital platforms (Cucuiat, 2018; Lin et al., 2024; Vaslin and Prié, 2025). However, when learning analytics is primarily oriented toward predicting grades, dropout, or final performance, this exploratory dimension becomes absorbed by performance-oriented variables and progressively loses value as an analytical object, despite evidence showing that distributed behavioral trajectories contain relevant information about participation and educational self-regulation (Li et al., 2024; Liu et al., 2026).

The problem addressed in this work emerges from this tension. Analyzing informal or exploratory patterns requires observing subtle behavioral traces, but these traces also increase the exposure of student data when centralized for model training. Consequently, the detection of informal learning is not presented as a definitive pedagogical classification, but rather as an operational representation derived from observable signals in VLE logs. The central question is not whether educational logs “prove” informal learning, but whether they allow reconstruction of behavioral patterns compatible with autonomous exploration and whether this representation can preserve analytical utility and interpretability under decentralized training conditions. This issue becomes increasingly relevant in privacy-aware educational artificial intelligence (AI) scenarios, where reducing direct exposure of behavioral logs and preserving explanatory consistency are becoming methodological constraints comparable in importance to predictive utility.

To address this problem, a framework integrating behavioral learning analytics, federated learning (FL), and explainable AI was developed (Ayeelyan et al., 2025). The study audited OULAD and EdNet, selecting OULAD as the primary corpus because it preserves explicit relationships among students, modules, presentations, VLE resources, activity types, and their relative temporal order. EdNet was used as a structural reference; however, its local partitions are primarily oriented toward question-solving sequences and do not provide a direct equivalent to non-evaluative navigation within the VLE environment. Using OULAD, 29,228 student–module–presentation analytical units were reconstructed from 10,900,970 interaction records, generating a behavioral layer based on resource diversity, revisits, temporal dispersion, navigation irregularity, and the proportion of non-evaluative activity.

The resulting research gap is methodological rather than purely algorithmic: existing studies rarely evaluate whether behaviorally interpretable exploratory representations can simultaneously retain utility, reduce direct exposure to raw data, and preserve explanatory structure under federated training. The methodological contribution of this study, therefore, consists in jointly evaluating three dimensions that are commonly analyzed separately: the stability of an exploratory behavioral proxy, its analytical utility under centralized and FL configurations, and the preservation of explanatory consistency. The informal/exploratory proxy was defined using a normalized score and a binary label based on the 75th percentile, avoiding reliance on final grades or academic performance as the primary target. Subsequently, Logistic Regression (LR) and Random Forest (RF) were evaluated as centralized models, while a compact MLP was trained under both centralized and FedAvg-based federated configurations. The federated simulation considered academic-module partitions, balanced random partitions, and a variant incorporating light Gaussian noise in the parameter updates (Chauhan et al., 2024). Explainability was evaluated through permutation importance, Overlap@10, and Spearman correlation between feature-importance rankings.

The study contributes a reproducible operationalization of informal/exploratory learning based on interpretable VLE trace indicators, evaluates its recoverability under centralized and federated configurations, quantifies reduced direct raw-data exposure and communication overhead under simulated FedAvg without claiming formal differential privacy, and examines whether explanatory rankings remain stable when model training is decentralized.

The results suggest that the reconstructed representation does not depend on an arbitrarily fragile decision boundary. The p70, p75, and p80 variants maintained agreement values close to 0.95 and Jaccard similarity values between approximately 0.80 and 0.83, indicating structural stability under moderate threshold variations. In the centralized scenario, LR achieved *F*1 = 0.977 and AUC-ROC = 1.000 in the operational configuration, while the reduced control maintained *F*1 = 0.905 and AUC-ROC = 0.991 after excluding the direct proxy components. This behavior suggests that the exploratory signal is distributed across the broader behavioral structure rather than being exclusively tied to the operational proxy formulation.

Under federated configurations, FedAvg by module achieved *F*1 = 0.671 and AUC-ROC = 0.886, values practically equivalent to those obtained by the centralized MLP. Simultaneously, the federated scenarios shared no raw interaction records under the exposure definition adopted in this study, although no formal differential privacy guarantees were claimed. The most relevant result was that decentralization did not substantially alter the dominant explanatory structure: the centralized MLP and FedAvg shared Overlap@10 = 1.000 and a Spearman correlation close to ρ = 0.985.

## 2 Literature review

Learning analytics has evolved from approaches centered primarily on performance monitoring toward frameworks capable of reconstructing behavioral patterns associated with participation dynamics, interaction continuity, and autonomous learning in virtual educational environments. Previous studies have shown that traces generated within VLE platforms contain structural information related to navigation, temporality, and collaborative interaction, enabling the identification of latent behavioral patterns beyond grades or direct evaluative outcomes (D'Aquin et al., 2018; Dawson et al., 2019). More recent research has expanded this perspective toward informal and exploratory learning processes, suggesting that distributed interaction with educational resources can reflect autonomous trajectories that remain partially invisible through conventional academic indicators (Cucuiat, 2018; Vaslin and Prié, 2025). Nevertheless, most existing approaches continue prioritizing predictive accuracy or engagement classification over the interpretable operationalization of exploratory behavioral patterns derived directly from educational traces.

In parallel, the increasing integration of AI into learning analytics systems has intensified concerns regarding privacy, student-data exposure, and the centralization of behavioral records (Agudo-Peregrina et al., 2012). Recent studies warn that analytics-based educational systems can reconstruct learning habits, temporal routines, and interaction preferences from seemingly innocuous traces (Joseph-Richard and Uhomoibhi, 2024; Khalil et al., 2025). This issue becomes especially relevant in AI-driven educational platforms, where behavioral records are continuously used for personalization, prediction, and pedagogical adaptation. In response, privacy-preserving learning analytics approaches have increasingly incorporated distributed learning mechanisms designed to reduce the direct transfer of sensitive educational data.

In this direction, FL has gained prominence as an alternative collaborative-training paradigm that does not require centralized raw data. The foundational work of Brendan McMahan et al. (2017) demonstrated that multiple clients can jointly train models by sharing parameter updates instead of the original data. More recent reviews have highlighted the expansion of FL into sensitive domains such as healthcare, IoT, cybersecurity, and education because of its capacity to reduce direct exposure of private information (Chaudhary et al., 2025; Yurdem et al., 2024).

Nevertheless, educational studies based on FL still present important limitations. First, most approaches focus almost exclusively on predictive performance metrics, such as accuracy or recommendation quality, without evaluating whether interpretable behavioral signals remain stable after distributed aggregation. Second, the non-IID heterogeneity characteristic of educational environments is frequently treated only as a convergence problem, ignoring that differences across courses, academic modules, and interaction patterns can substantially alter the explanatory structure of the resulting models.

At the same time, recent literature on educational AI has emphasized the importance of incorporating explainability and transparency into analytical systems applied to learning processes. Explainable AI (XAI) has been used to improve trust, pedagogical interpretability, and understanding of automated decisions in educational platforms (Yang et al., 2023; Zhang et al., 2021). However, most XAI-oriented studies remain centered on fully centralized scenarios and evaluate interpretability independently of privacy constraints or decentralization mechanisms. This separation constitutes a relevant methodological limitation because preserving predictive utility under FL does not necessarily imply preserving explanatory consistency (Tao et al., 2025). Federated aggregation can modify feature-importance rankings, redistribute behavioral signals across clients, or alter dominant explanatory variables even when global predictive metrics remain relatively stable.

Another important limitation concerns the operationalization of informal or exploratory learning. Although numerous studies address autonomous learning, self-directed learning, and exploratory behavior from conceptual perspectives, relatively few define reproducible behavioral proxies derived directly from VLE traces (Lin et al., 2024; Liu et al., 2026). Many existing approaches rely on surveys, institutional labels, or variables that are difficult to generalize across educational platforms. Consequently, the relationship among exploratory behavioral patterns, educational privacy, and distributed learning architectures remains insufficiently formalized within the learning analytics literature.

Given these limitations, the present study integrates behavioral learning analytics, FL, and XAI into a unified analytical framework for operationalizing informal and exploratory behavioral patterns. Unlike previous approaches focused primarily on predictive accuracy, this framework simultaneously evaluates preservation of analytical utility, reduction of raw-data exposure, and explanatory consistency under both centralized and federated configurations.

## 3 Materials and methods

### 3.1 Dataset audit and behavioral representation

The study was structured around reconstructing behavioral patterns from traces recorded in VLEs. Initially, two large-scale educational datasets, OULAD and EdNet, were audited. Although both datasets contain extensive records of student interactions, substantial structural differences emerged during the audit regarding event organization, temporal granularity, and the ability to reconstruct distributed behavioral trajectories. OULAD preserves explicit relationships among students, modules, academic presentations, VLE resources, and heterogeneous activity types within an integrated tabular schema (Choi et al., 2025). In contrast, EdNet is primarily organized around question-solving sequences distributed across fragmented user-specific files, prioritizing evaluative events over broader educational navigation patterns.

The audit evaluated structural coverage, identifier consistency, temporal availability, activity diversity, and the capacity to reconstruct longitudinal behavioral traces. OULAD simultaneously preserved activities associated with navigation (subpage, homepage), collaborative interaction (forumng), content interaction (oucontent), and resource access (resource, url), enabling reconstruction of behavioral trajectories extending beyond strictly evaluative sequences. Although EdNet maintained a substantially larger volume of records, most events were concentrated around response and evaluation activities, limiting the consistent recovery of exploratory behavioral patterns distributed throughout the VLE environment.

Figure 1 presents the complete methodological workflow adopted in the study. The pipeline integrates dataset auditing, reconstruction of behavioral traces, feature-layer generation, construction of the informal/exploratory proxy, centralized and federated modeling, and joint evaluation of analytical utility, privacy, and explainability. The modular organization of the workflow preserved consistency between centralized and federated scenarios while maintaining traceability of the transformations applied to the original educational interactions.

**Figure 1:**
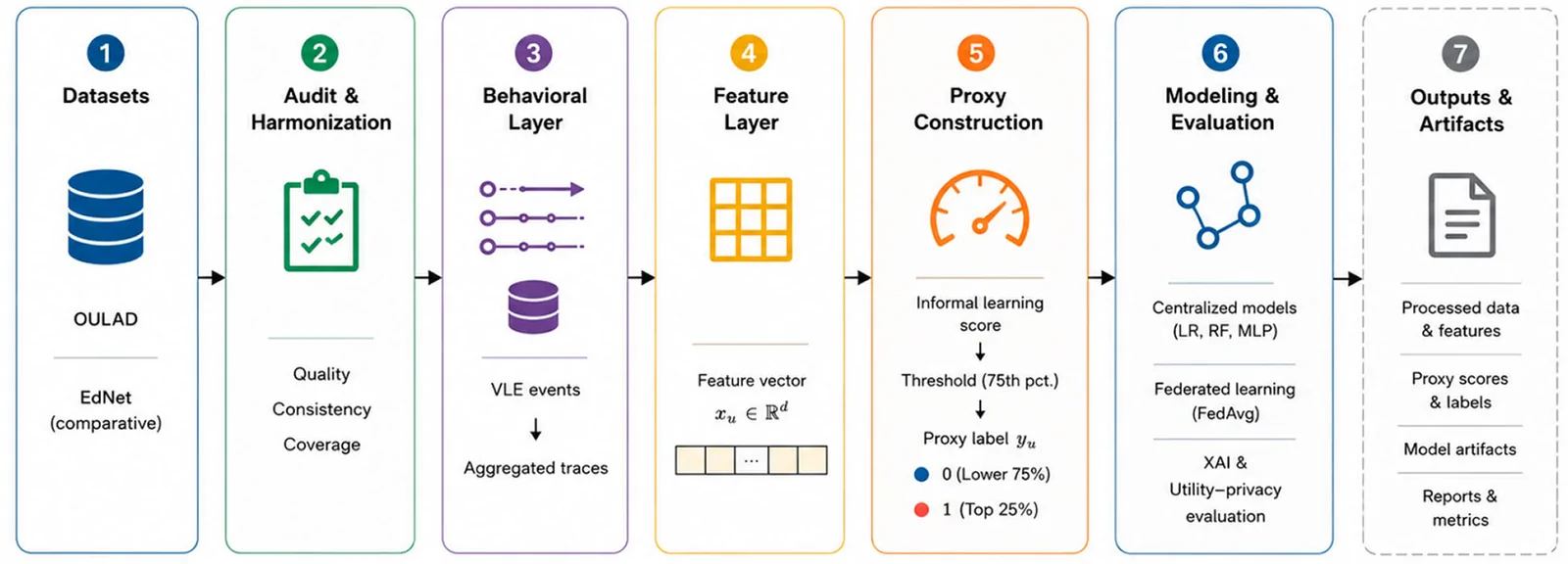
Overall methodological framework integrating dataset audit, behavioral feature construction, informal/exploratory proxy generation, centralized and federated modeling, and explainability-oriented utility–exposure evaluation.

The analytical unit was defined at the student–module–presentation level to preserve continuity within each educational trajectory. Equation 1 formalizes the structure of the analytical unit adopted in the study:


$$\begin{array}{l} u_{i}=\left(s_{i},m_{i},p_{i}\right),\;i=1,\ldots ,N \end{array}$$ (1)

where *s*_*i*_ represents the student identifier, *m*_*i*_ the academic module, and *p*_*i*_ the corresponding academic presentation. Each analytical unit *u*_*i*_ groups the complete sequence of VLE interactions associated with a student within a specific presentation.

The reconstructed traces were transformed into aggregated behavioral representations composed of heterogeneous events, including navigation, content interaction, resource transitions, revisits, and collaborative activities. Equation 2 defines the behavioral feature vector used to represent each educational trajectory:


$$\begin{array}{l} x_{u}={\left[x_{u1},x_{u2},\ldots ,x_{ud}\right]}^{T}\in {\mathbb{R}}^{d} \end{array}$$ (2)

where each component corresponds to an aggregated behavioral descriptor derived from VLE traces. The behavioral layer integrated variables associated with temporal dispersion, navigation irregularity, resource diversity, interaction continuity, revisits, and the proportion of non-evaluative activity.

Furthermore, the behavioral representation functioned as an intermediate abstraction layer between raw VLE events and the subsequent stages of proxy construction and analytical modeling. This separation enabled decoupling behavioral-feature extraction from specific centralized or FL configurations while preserving compatibility between the two experimental scenarios.

Resource diversity was modeled using the normalized entropy of the educational resources accessed within each trajectory. Let *p*_*ur*_ denote the proportion of interactions associated with resource *r* within analytical unit *u*. Equation 3 defines the normalized entropy-based diversity measure adopted in the study:


$$\begin{array}{l} {\;H}_{u}=-\underset{r\in R_{u}}{\sum }p_{ur}\log\left(p_{u\text{r}}\right),\\ \text{resource\_diversity}\left(u\right)=\frac{H_{u}}{\log\left(\max\left(|R_{u}|,2\right)\right)} \end{array}$$ (3)

### 3.2 Informal learning proxy construction

The target variable of the study was defined as a behavioral proxy representing informal and exploratory behavior derived from observable regularities in VLE traces. The formulation deliberately avoids using final grades, assessment scores, or academic outcomes as predictive targets. Instead, the proxy was designed to represent trajectories characterized by relatively higher levels of autonomous exploration, non-evaluative activity, navigation diversity, revisits, and temporally distributed interaction patterns.

For each student–module–presentation analytical unit, five primary behavioral components were extracted: proportion of non-evaluative activity, resource diversity, revisit rate, temporal dispersion, and navigation irregularity. These components were selected because they capture complementary dimensions of exploratory behavior, including interaction beyond evaluative activities, distributed consultation of educational materials, return to previously visited resources, continuity of participation throughout the course, and variability in navigation paths. Conceptually, the proxy treats exploratory behavior as a trace-based pattern of autonomous engagement rather than as a latent psychological state: non-evaluative activity indicates interaction not directly driven by assessment completion, resource diversity captures breadth of material consultation, revisits indicate iterative return to prior resources, temporal dispersion reflects sustained engagement across the presentation, and navigation irregularity captures non-linear movement across VLE activity categories.

Behavioral variables were normalized using Min–Max scaling to prevent differences in magnitude from dominating the composite exploratory score. Equation 4 defines the normalization procedure adopted for each behavioral component:


$$\begin{array}{l} {\tilde{x}}_{uj}=\frac{x_{uj}-\min\left(x_{j}\right)}{\max\left(x_{j}\right)-\min\left(x_{j}\right)+\epsilon } \end{array}$$ (4)

where *x*_*uj*_ represents the value of feature *j* for analytical unit *u*, while min(*x*_*j*_) and max(*x*_*j*_) correspond to the minimum and maximum values observed for feature *j* within the analytical sample. The constant ϵ was incorporated to prevent division-by-zero conditions during normalization.

The informal/exploratory score was computed as a weighted combination of the normalized behavioral components. Equation 5 defines the composite exploratory score used in the study:


$$\begin{array}{l} z_{u}=\underset{j\in B}{\sum }w_{j}{\tilde{x}}_{uj},\;\underset{j\in B}{\sum }w_{j}=1 \end{array}$$ (5)

where *B* corresponds to the set of behavioral components included in the proxy, and *w*_*j*_ represents the weight assigned to behavioral dimension *j*. In the baseline configuration, equal weights were adopted as a conservative and transparent design choice: the objective was not to optimize the proxy for a downstream classifier, but to avoid privileging any single behavioral dimension without external pedagogical labels or expert-elicited weights. This choice makes the proxy reproducible and auditable, while leaving alternative weighting strategies as a sensitivity dimension rather than as part of the primary definition.

The binary proxy label was subsequently defined using a percentile threshold over the exploratory-score distribution. Equation 6 formalizes the labeling criterion employed in the study:


$$\begin{array}{l} y_{u}=𝕀\left(z_{u}\geq Q_{0.75}\left(z\right)\right) \end{array}$$ (6)

where *Q*_0.75_(*z*) represents the 75th percentile of the informal/exploratory score distribution. Analytical units located within the upper quartile were labeled as high-intensity exploratory trajectories, whereas the remaining units were assigned to the low/middle exploratory class. This operational definition was implemented through Algorithm 1, which integrates behavioral-signal extraction, normalization, score construction, and generation of reproducible proxy labels from aggregated VLE traces.

Algorithm 1 Informal/exploratory proxy construction. 
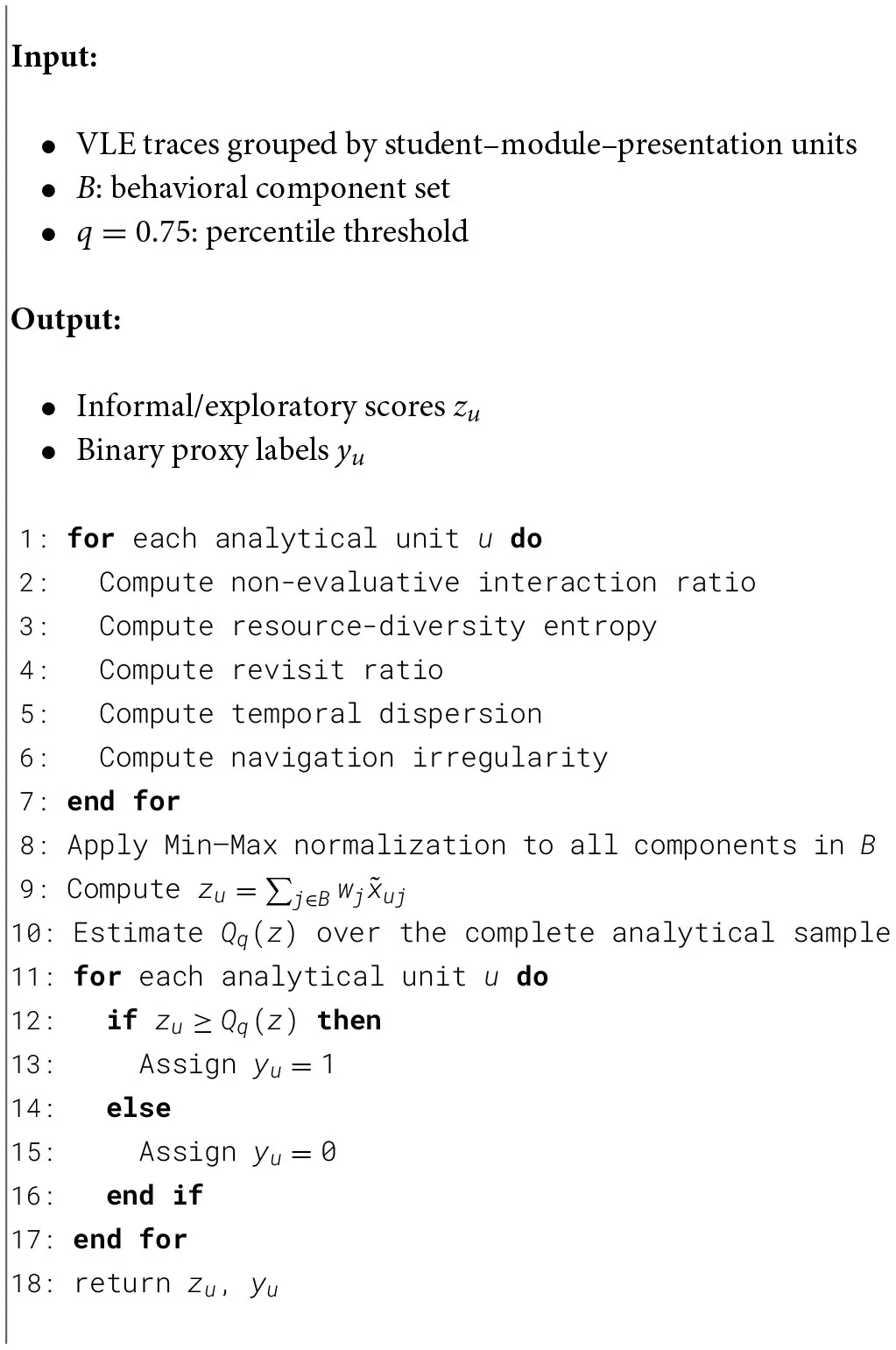

The sensitivity of the proxy was evaluated through controlled variations of both the percentile threshold and the set of behavioral components included in the formulation. The p70, p75, and p80 configurations enabled analysis of structural stability under moderate variations in the high-intensity exploratory boundary. Simultaneously, alternative configurations incorporating forum activity, activity outside the expected temporal window, and expanded behavioral components allowed examination of whether the composition of the exploratory class depended on specific behavioral signals. Stability was quantified using overall agreement and Jaccard similarity between the high-intensity exploratory class and the baseline configuration.

### 3.3 Centralized and federated learning setup

The comparative analysis was structured around two complementary scenarios: centralized learning and simulated FL. The centralized configuration established a benchmark for analytical utility under full access to the aggregated behavioral layer. In contrast, the federated configuration evaluated preservation of analytical utility and explanatory consistency under local data separation across educational clients.

LR and RF were adopted as centralized baseline models focused on interpretability and recovery of the informal/exploratory proxy from aggregated behavioral signals. LR was selected because of its ability to model transparent linear relationships between behavioral variables and the proxy label, whereas RF enabled capture of secondary nonlinear relationships without introducing large-scale deep architectures.

The federated scenario was constructed using a compact multilayer perceptron (MLP) with two hidden layers to preserve comparability between centralized training and FedAvg-based distributed training. The architecture was intentionally maintained at low complexity to avoid excessive dependence on over-parameterization while preserving traceability of the behavioral signals used during training. Equation 7 formalizes the centralized optimization process:


$$\begin{array}{l} \theta^{*}=\arg\underset{\theta }{\min}\frac{1}{N}\overset{N}{\underset{i=1}{\sum }}L\left(f_{\theta }\left(x_{i}\right),y_{i}\right)+\lambda \Omega \left(\theta \right) \end{array}$$ (7)

where *x*_*i*_ corresponds to the behavioral vector associated with analytical unit *i*, *y*_*i*_ denotes the informal/exploratory proxy label, L represents the binary loss function, and Ω(θ) corresponds to the regularization term applied to the trainable model parameters.

The federated simulation employed FedAvg as the distributed aggregation mechanism. Analytical units were partitioned using two complementary strategies: academic-module partitioning and balanced random partitioning. The first strategy approximated the non-IID educational heterogeneity associated with curricular and behavioral differences across academic modules, whereas the second functioned as a structural control under comparatively homogeneous client distributions. Equation 8 defines the global aggregation process used during federated training:


$$\begin{array}{l} \theta^{\left(r+1\right)}=\overset{K}{\underset{j=1}{\sum }}\frac{n_{j}}{N}\theta_{j}^{\left(r+1\right)} \end{array}$$ (8)

where $\theta_{j}^{\left(r+1\right)}$ represents the parameters updated by client *j* during federated round (*r*+1), *n*_*j*_ denotes the number of local samples associated with client *j*, and *N* corresponds to the total number of analytical units participating in the global aggregation process. In the simulated setting, all clients participated in every communication round. The academic-module partition produced seven clients corresponding to OULAD modules, whereas the balanced random partition produced 10 clients. Client-level sample counts, positive-class rates, and local performance were retained to characterize non-IID heterogeneity and class imbalance across clients.

In addition to the baseline federated configuration, a variant incorporating slight Gaussian perturbations into the local parameter updates was introduced to evaluate the sensitivity of the exchanged parameters to noise. This configuration was not intended as a formal differential-privacy mechanism, but rather as a lightweight perturbation constraint applied to the updates shared during the FedAvg aggregation process.

The approximate communication overhead associated with federated training was estimated using the formulation defined in Equation 9:


$$\begin{array}{l} \text{CommOverhead}=R\cdot K\cdot |\theta |\cdot b \end{array}$$ (9)

where *R* represents the number of federated rounds, *K* the number of participating clients, |θ| the total number of exchanged model parameters, and *b* the number of bytes used to serialize each parameter during communication.

The experimental configuration preserved reproducible separation between training and testing through stratified partitioning. Evaluation metrics included Accuracy, Precision, Recall, F1-score, and AUC–ROC to assess overall analytical utility, together with local per-client metrics designed to characterize federated heterogeneity. Table 1 summarizes the principal configurations adopted in both the centralized models and the evaluated federated scenarios.

**Table 1:** Compact configuration of centralized and federated models.

Component	Parameter	Value
Logistic regression	Penalty	L2
Logistic regression	Maximum iterations	2,000
Logistic regression	Class balance	Balanced
Random forest	Trees	300
Random forest	Maximum depth	12
Random forest	Minimum samples per leaf	10
MLP	Hidden layers	32–16
MLP	Activation	ReLU
MLP	Output layer	Sigmoid
MLP	Trainable parameters	1,729
FedAvg	Federated rounds	35
FedAvg	Local epochs	3
FedAvg	Learning rate	0.015
FedAvg	Client participation	100% per round
FedAvg	Clients	Module/random-10
FedAvg	Local update rule	Full-batch gradient descent
FedAvg	Early stopping/LR schedule	Not used
General	Random seed	42
General	Test split	30% stratified
MLP	Threshold selection	Train-set F1 calibration
FedAvg + noise	Noise standard deviation	0.002

### 3.4 Privacy, explainability, and consistency evaluation

The evaluation framework was structured around three complementary dimensions: reduction of direct raw-data exposure, preservation of analytical utility, and explanatory consistency between centralized and federated configurations. The objective was not to model formal differential privacy or cryptographic anonymization mechanisms, but rather to quantify the extent to which local data separation preserves predictive utility and interpretive stability during distributed training.

Reduction of direct raw-data exposure was estimated using a privacy-aware proxy defined as the proportion of raw records that remained locally stored during federated training. Let *R*_*c*_ denote the number of raw records available under centralized training and *R*_*f*_ the number of raw records shared during the federated process. Equation 10 defines the privacy proxy adopted in the study:


$$\begin{array}{l} \text{PrivacyGain}=1-\frac{R_{f}}{R_{c}} \end{array}$$ (10)

where values approaching 1 indicate minimal direct exposure of raw data during distributed training. In the evaluated federated configurations, only parameter updates were exchanged, while the original behavioral traces remained locally stored at each client.

Analytical utility was evaluated using Accuracy, Precision, Recall, F1-score, and AUC–ROC over stratified test partitions. In addition to global metrics, local per-client metrics were computed to characterize variability associated with non-IID heterogeneity under both academic-module and balanced-random partitioning strategies.

Explanatory consistency between centralized and federated models was estimated through permutation-based feature importance computed within the same behavioral feature space. This procedure enabled direct comparison of importance rankings across equivalent architectures without dependence on client-specific interpretation mechanisms. To quantify preservation of the dominant explanatory structure, the overlap between feature rankings was measured using the Overlap@*k* metric defined in Equation 11:


$$\begin{array}{l} \text{Overlap@k}\left(A,B\right)=\frac{\left|Top_{k}\left(A\right)\cap Top_{k}\left(B\right)\right|}{k} \end{array}$$ (11)

where *Top*_*k*_(*A*) and *Top*_*k*_(*B*) represent the sets of the *k* most important behavioral variables obtained from the compared models.

The relative stability of the explanatory ordering was additionally quantified using Spearman correlation between complete feature-importance rankings. Equation 12 formalizes the correlation measure employed in the analysis:


$$\begin{array}{l} \rho =1-\frac{6\sum_{i=1}^{n}d_{i}^{2}}{n\left(n^{2}-1\right)} \end{array}$$ (12)

where *d*_*i*_ represents the difference in ranking position associated with behavioral feature *i*, and *n* corresponds to the total number of behavioral features included in the comparison.

The explanatory comparison was conducted primarily between the centralized MLP and the FedAvg configuration partitioned by academic module, since both models share the same architecture and differ only in their training procedures. LR was incorporated as an additional linear reference model to examine whether observed variations were associated with the model family itself rather than exclusively with the federated-learning process.

The relative utility loss under federation was estimated through direct comparison between the global performance metrics of the centralized MLP and those obtained by the equivalent FedAvg variants. This procedure enabled isolation of decentralization-associated effects from variations introduced by architectural differences between models. Simultaneously, local per-client metrics enabled characterization of federated stability under heterogeneous distributions of educational behavior.

### 3.5 Reproducibility and computational configuration

The experimental implementation was developed in Python 3.11 (Python Software Foundation, Beaverton, OR, USA) within a reproducible computational environment using NumPy, Pandas, Scikit-learn, and SciPy. All experiments were executed on a workstation equipped with a multi-core CPU, more than 32 GB of RAM, and optional GPU acceleration for MLP training. The computational configuration was intentionally designed to remain lightweight, prioritizing methodological stability and behavioral traceability over intensive optimization or deep over-parameterization.

Reproducibility was preserved by explicitly fixing pseudo-random seeds at all critical stages of the analytical pipeline, including stratified partitioning, model initialization, federated training, and Gaussian perturbation of local updates. Training and testing partitions were maintained constant across experimental runs to avoid variability between successive executions. Similarly, federated partitions by academic module and balanced random partitions were serialized before training to preserve consistency across the compared 'scenarios.

The framework was implemented following a modular architecture that separated dataset auditing, behavioral extraction, proxy construction, centralized training, federated simulation, and explainability analysis into independent components. This modular design enabled reuse of the same behavioral representation across all experimental scenarios without modifying the transformations applied to the original VLE traces. Furthermore, separation between processing modules facilitated comparison between centralized and federated configurations while preserving equivalence across the feature space, evaluation metrics, and analytical procedures.

The approximate computational complexity of federated training was dominated by the number of aggregation rounds, participating clients, local epochs, local sample counts, and trainable parameters exchanged during the FedAvg process. Equation 13 defines the aggregate complexity approximation adopted in the study:


$$\begin{array}{l} O\left(R\cdot \overset{K}{\underset{j=1}{\sum }}E_{\text{local}}\cdot n_{j}\cdot |\theta |\right) \end{array}$$ (13)

where *R* represents the number of federated rounds, *K* the number of participating clients, *E*_local_ the number of local epochs, *n*_*j*_ the number of local samples in client *j*, and |θ| the total number of trainable parameters of the MLP architecture.

The explainability analysis based on permutation importance exhibited computational complexity dependent on both the number of behavioral features and the size of the evaluated analytical sample. Equation 14 formalizes the corresponding complexity approximation:


$$\begin{array}{l} O\left(F\cdot N\right) \end{array}$$ (14)

where *F* corresponds to the number of behavioral features and *N* to the total number of analytical units included in the importance-estimation process.

## 4 Results

### 4.1 Suitability of the dataset and behavioral analysis layer

The structural audit revealed substantial differences between OULAD and EdNet partitions in terms of organization, granularity, and observable behavioral density. Although EdNet-KT1 exhibited the largest estimated record volume, with approximately 7.03 × 10^7^ rows distributed across 748,230 CSV files, most interactions were concentrated around question-solving sequences and response-associated events. In contrast, OULAD consolidated 10,900,970 interaction records into seven integrated tabular files while preserving explicit relationships among students, VLE resources, temporal activity, and interaction types. Table 2 summarizes the structural differences identified during the dataset audit.

**Table 2:** Suitability of the audited datasets and structural availability.

Dataset/section	CSV	Profiling	MB	Exact rows	Estimated rows
EdNet-KT1/KT1	748,230	Sampled_first_pass	2,656.009	N/A	70,258,797
EdNet-KT/KT1	36,079	Sampled_first_pass	274.027	N/A	7,971,149
EdNet-KT/KT2	23	Sampled_first_pass	0.064	N/A	1,540
OULAD	7	Full	442.855	10,900,970	N/A

Figure 2A shows that total dataset scale was not directly associated with the behavioral richness available for exploratory analysis. EdNet-KT1 reached the largest scale in terms of both storage size and estimated number of rows; however, this was accompanied by extreme fragmentation into user-specific files. In contrast, OULAD, despite occupying an intermediate position in terms of total scale, preserved a substantially more integrated structure that enabled reconstruction of continuous interaction trajectories within the VLE environment. This structural difference became particularly evident in the recovery of non-evaluative activity and distributed navigation across educational resources.

**Figure 2:**
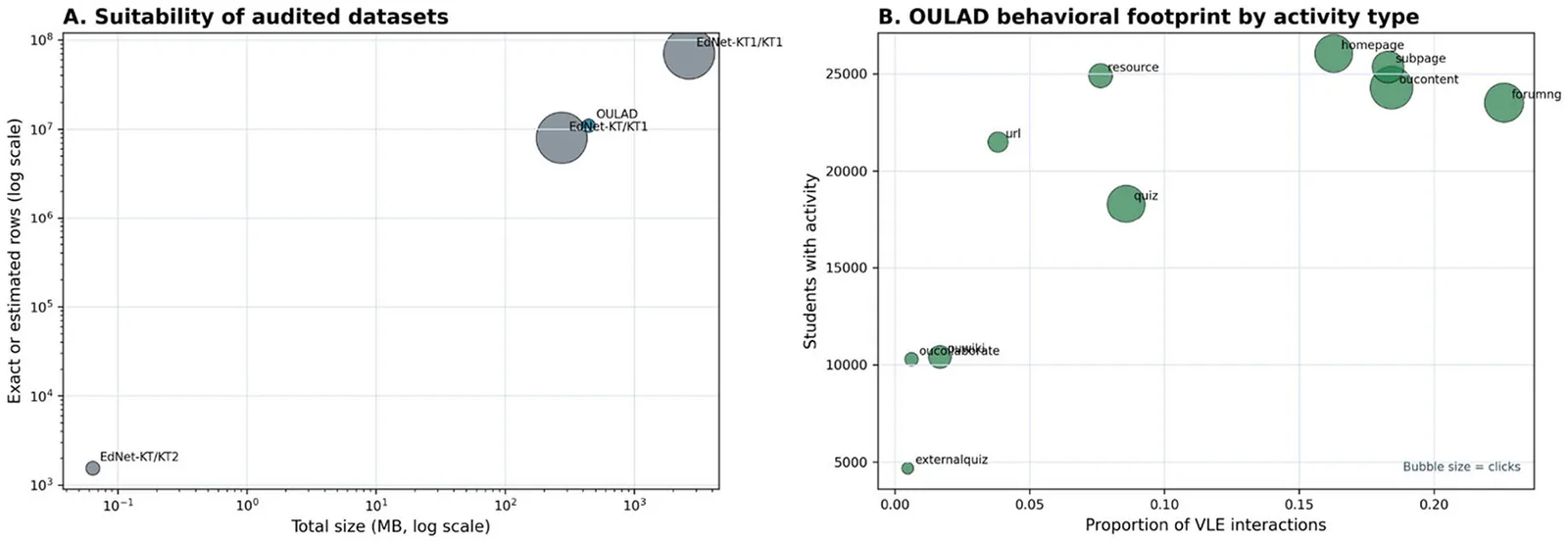
Suitability of the datasets and behavioral footprint of OULAD. **(A)** Relationship between size, number of rows, and number of files in the audited sections. **(B)** Distribution of activity types in the VLE according to the proportion of interactions, the number of students reached, and the volume of clicks.

The distribution of activity types observed in OULAD showed that educational interaction was not restricted to evaluative components. As illustrated in Figure 2B, the categories forumng, oucontent, subpage, and homepage collectively accounted for a substantial proportion of interactions and broad student coverage. The forumng category represented approximately 22.6 % of the recorded interactions, while oucontent exhibited the highest relative click volume among content-oriented activities. These results indicate that a considerable fraction of student activity was distributed across material consultation, resource navigation, and participation in interactive educational spaces beyond direct assessment completion.

Activities associated with educational resources (resource, url) exhibited comparatively lower click density but broad population coverage, suggesting cross-sectional consultation patterns distributed across the student population. In contrast, categories such as externalquiz and questionnaire simultaneously showed low interaction frequency and limited population reach, indicating more localized participation patterns within the VLE structure. This asymmetry across activity types suggests that the observed behavioral footprint did not depend on a single dominant interaction component, but rather on a heterogeneous combination of navigation, content interaction, and transitions among educational resources.

The consolidated behavioral layer reconstructed at the student–module–presentation level also exhibited substantial dispersion in both interaction intensity and structural organization. The average number of interactions per analytical unit reached 364.56, with certain trajectories exceeding 3,000 interactions in specific academic modules. However, interaction intensity did not behave equivalently to behavioral exploration. Some trajectories characterized by high click volume remained concentrated within relatively small subsets of resources, whereas other profiles with lower overall activity exhibited substantially greater diversity in navigation and transitions across educational content. This difference suggests that exploratory behavior emerges primarily as a structural interaction configuration rather than solely as an increase in activity frequency within the VLE.

### 4.2 Proxy construction and methodological sensitivity

The quantitative sensitivity of the proxy remained stable under moderate variations in the percentile threshold. As presented in Table 3, the configurations based on the 70th, 75th, and 80th percentiles maintained agreement values close to 0.95 relative to the baseline 75th-percentile configuration. Simultaneously, the overlap of the high-intensity exploratory class remained between 0.800 and 0.833 according to the Jaccard similarity metric. The progressive reduction in the number of trajectories classified as exploratory, decreasing from 8,769 cases at the 70th percentile to 5,846 at the 80th percentile, did not produce abrupt structural reorganizations in the composition of the high-intensity class. This behavior suggests that trajectories located within the upper region of the exploratory-score distribution remained comparatively stable under moderate variations in the cutoff threshold.

**Table 3:** Sensitivity of the informal/exploratory proxy under alternative thresholds and component configurations.

Condition	Agreement	Jaccard	Upper class
Baseline (five components, p70)	0.950	0.833	8,769
Baseline (five components, p75)	1.000	1.000	7,307
Baseline (five components, p80)	0.950	0.800	5,846
Baseline + forum ratio (p75)	0.897	0.659	7,307
Baseline + out-of-week activity (p75)	0.837	0.509	7,307
Expanded proxy (seven components, p75)	0.824	0.479	7,307

The variants constructed through structural expansion of the proxy produced substantially greater modifications in stability than those generated by percentile variations alone. The incorporation of forum_ratio reduced the Jaccard similarity of the high-intensity exploratory class to 0.659, whereas the plus_out_of_expected_week_p75 configuration decreased to 0.509. The expanded seven-component variant exhibited the lowest relative stability, reaching Agreement = 0.824 and Jaccard = 0.479. This pattern demonstrates that the internal composition of the exploratory class was considerably more sensitive to modifications in the conceptual structure of the proxy than to moderate variations in the operational percentile threshold.

Differences among configurations additionally revealed that behavioral dimensions did not contribute equivalent information regarding exploratory activity. The inclusion of forum interaction produced partial structural modifications while preserving relatively high overlap with the baseline configuration. In contrast, incorporation of activity outside the expected temporal window displaced a substantial fraction of the trajectories previously classified as exploratory. This behavior suggests that out-of-sequence temporal dynamics constitute a behavioral dimension partially distinct from the autonomous navigation patterns reflected through resource diversity, revisits, and interaction continuity.

Figure 3A shows that the proxy did not produce an abrupt natural separation between exploratory and non-exploratory profiles. The score distribution remained strongly concentrated within intermediate-to-high values, particularly between approximately 0.68 and 0.76, whereas extremely low-score trajectories were substantially less frequent. The 70th, 75th, and 80th percentiles were all located within a continuous density region without visible structural discontinuities in the observed behavioral distribution.

**Figure 3:**
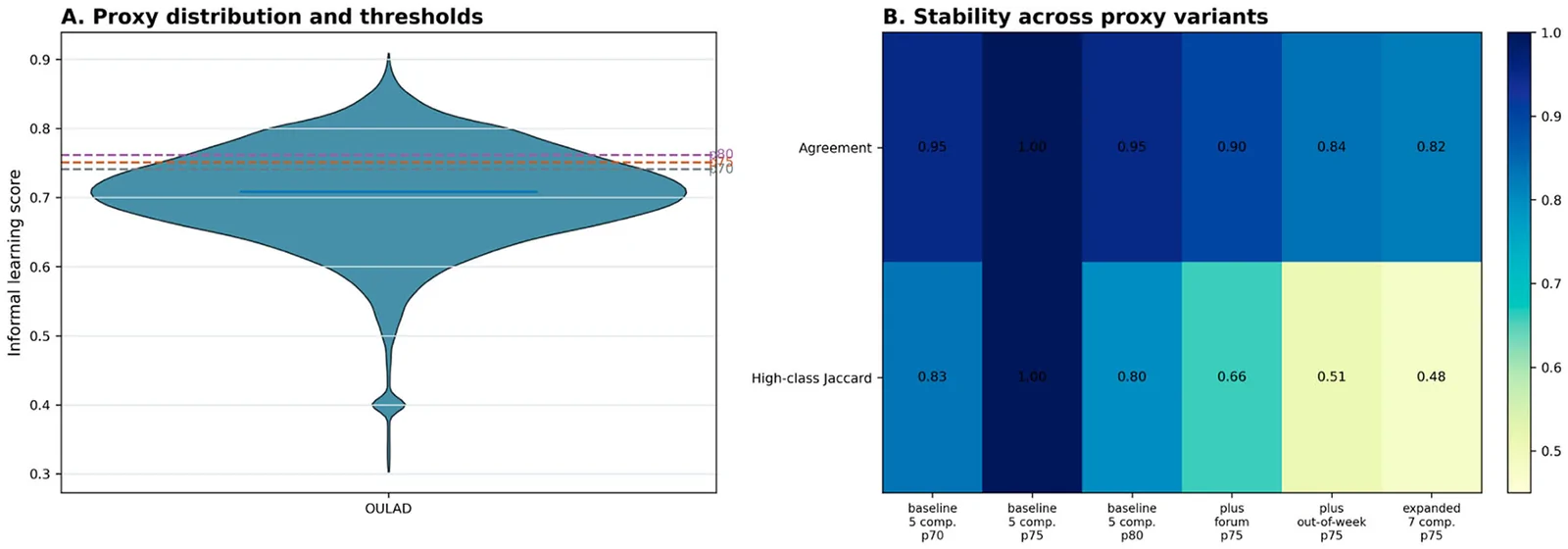
Distribution and sensitivity of behavioral proxy. **(A)** Distribution of the informal/exploratory score with the p70, p75, and p80 thresholds. **(B)** Stability of the proxy variants against the baseline p75.

Figure 3B complements these results by showing that configurations based exclusively on percentile modifications preserved high levels of agreement and structural similarity relative to the baseline configuration. In contrast, variants associated with expansion of the behavioral components progressively reduced both overall agreement and overlap with the high-intensity exploratory class. The most pronounced degradation was observed in the expanded seven-component configuration, where similarity relative to the baseline decreased below 0.50 according to the Jaccard similarity metric. This reduction indicates that simultaneous incorporation of additional temporal and social dimensions substantially reorganized the structure of the trajectories classified as exploratory.

The stability observed across the p70–p80 configurations additionally revealed that the behavioral core of the proxy remained dominated by persistent regularities associated with navigation and interaction continuity. Trajectories consistently classified as exploratory across percentile configurations simultaneously maintained high resource diversity, frequent revisits, and substantial temporal dispersion. In contrast, the expanded configurations incorporated profiles characterized by more specific temporal or social patterns, altering the original composition of the exploratory class without necessarily modifying the overall interaction intensity within the VLE environment.

### 4.3 Centralized analytical utility

Centralized proxy retrieval demonstrated high analytical consistency even under reduced-variable configurations. As presented in Table 4, LR achieved the highest performance on the complete operational dataset, reaching Accuracy = 0.988, *F*1 = 0.977, and AUC-ROC = 1.000. This near-perfect result should be interpreted as an operational upper-bound for recovering a transparent additive proxy from behaviorally related variables, rather than as evidence of externally validated prediction of informal learning. RF also maintained strong analytical performance, although lower than that achieved by the linear model, with *F*1 = 0.920 and AUC-ROC = 0.993. The performance difference observed between the two models suggests that the principal structure of the proxy can be reconstructed from comparatively stable relationships among the behavioral variables included in its operational formulation.

**Table 4:** Centralized analytical performance under operational and reduced-control configurations.

Model	Accuracy	Precision	Recall	F1	AUC-ROC
Operational LR	0.988	0.956	0.999	0.977	1.000
Operational RF	0.959	0.887	0.956	0.920	0.993
Reduced LR	0.949	0.852	0.965	0.905	0.991
Reduced RF	0.933	0.829	0.922	0.873	0.983

The behavior of the reduced-control configuration proved particularly relevant for evaluating direct dependence on the original proxy components. After excluding variables explicitly associated with the operational formulation of the proxy, LR maintained *F*1 = 0.905 and AUC-ROC = 0.991, while RF preserved *F*1 = 0.873 and AUC-ROC = 0.983. Although the observed degradation was noticeable, the behavioral signal did not collapse under the reduced configuration. This behavior suggests that the exploratory structure remained partially distributed across indirect variables associated with temporal continuity, resource transitions, interaction intensity, and broader navigation patterns within the VLE environment.

The relative performance reduction additionally revealed differences between the evaluated model families. LR exhibited an approximate 7.4% reduction in *F*1 between the operational and reduced configurations, whereas RF showed an approximate 5.1% decrease. Although RF degraded proportionally less, its absolute performance remained lower in both evaluated scenarios. This pattern is consistent with the construction of the proxy itself, which combines normalized behavioral components through an additive score. LR therefore aligns closely with the geometry of the operational label, whereas RF captures complementary nonlinear interactions that are informative but not dominant for this specific recovery task.

Figure 4A shows that differences between *F*1 and AUC-ROC remained comparatively stable across the evaluated models, particularly under the operational configurations. LR simultaneously achieved the highest *F*1 and the strongest ROC separation, positioning the model in the upper region of the comparison space. RF preserved high discrimination capacity, although with overall performance shifted toward comparatively lower levels. Separation between models became more pronounced under the reduced configurations, where removal of direct proxy components increased dependence on secondary behavioral signals.

**Figure 4:**
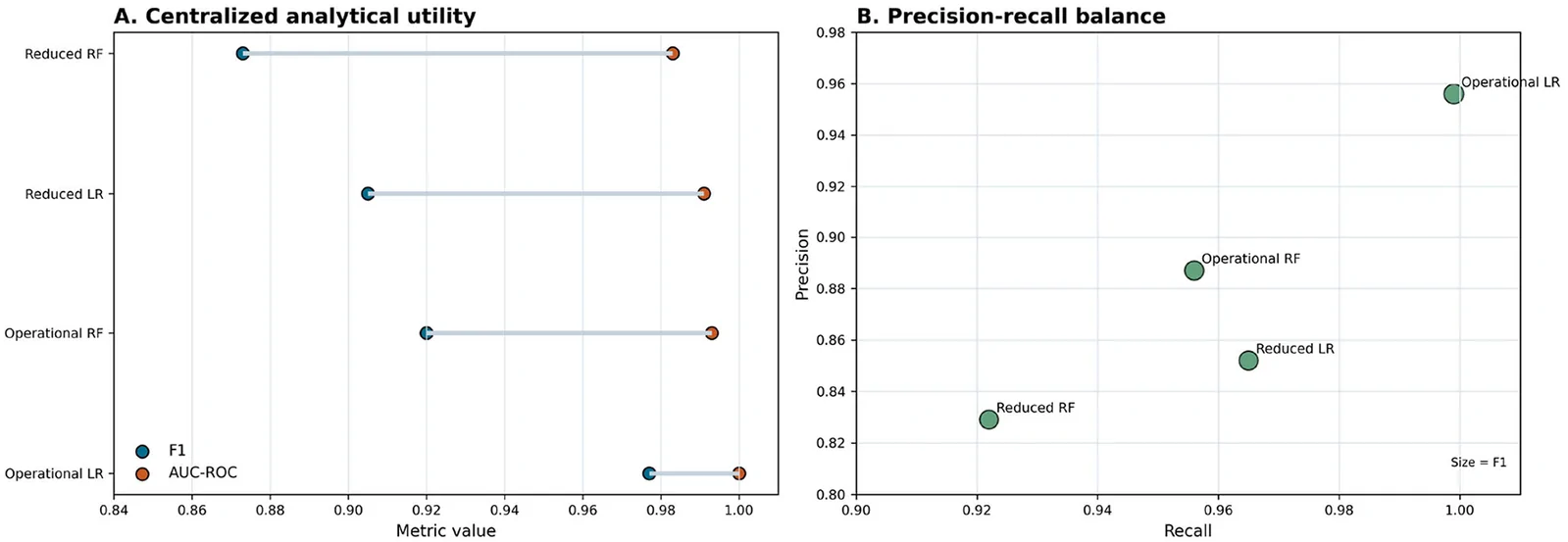
Centralized analytical utility. **(A)** Comparison between F1 and AUC-ROC in centralized models. **(B)** Balancing precision-recall, with spot size proportional to F1.

The accuracy–recall relationship illustrated in Figure 4B revealed particularly concentrated behavior for the operational LR configuration, which simultaneously occupied the region associated with maximum accuracy and maximum recall. Operational RF also maintained a high balance between both metrics, although shifted toward comparatively lower accuracy values. Under the reduced configurations, both models experienced simultaneous decreases in accuracy and recall while preserving a consistent recovery structure for the exploratory class. The persistence of recall values above 0.92, even under reduced-control conditions, indicates that a substantial proportion of the exploratory trajectories remained identifiable after removal of the components directly associated with the operational proxy formulation.

### 4.4 Federated utility and reduced data exposure

The comparison between the centralized MLP and the FedAvg variants showed near-complete preservation of analytical utility under simulated decentralized-training conditions. As presented in Table 5, the FedAvg module configuration achieved *F*1 = 0.671 and AUC-ROC = 0.886, reproducing the performance obtained by the equivalent centralized MLP after numerical rounding. Random FedAvg maintained the same overall analytical behavior, while the FedAvg module + noise configuration achieved *F*1 = 0.673 and AUC-ROC = 0.887. The differences observed among the federated configurations remained minimal in terms of both ROC discrimination and recovery of the exploratory class. At the same time, the federated scenarios introduced only communication overhead associated with parameter exchange, while preserving local separation of the original behavioral traces throughout distributed training.

**Table 5:** Trade-off between analytical utility and communication in federated configurations.

Scenario	F1	AUC-ROC	Updates	Communication (MB)
Central MLP	0.671	0.886	0	0.000
FedAvg module	0.671	0.886	245	1.616
Random FedAvg	0.671	0.886	350	2.308
FedAvg module + noise	0.673	0.887	245	1.616

The stability of *F*1 and AUC-ROC between the centralized scenario and the federated variants indicates that decentralization did not produce observable degradation in the MLP's ability to recover the exploratory proxy. This behavior remained consistent even under structurally different partitioning strategies. The random FedAvg configuration increased the number of exchanged updates to 350 and raised the approximate communication overhead to 2.308 MB while preserving the same overall analytical utility. In contrast, academic-module partitioning required fewer updates and lower communication overhead while maintaining equivalent aggregate performance.

The FedAvg module + noise configuration exhibited particularly stable behavior under minor perturbations of local parameter updates. Incorporation of Gaussian noise did not produce visible reductions in either *F*1 or AUC-ROC relative to the baseline federated scenario. The variation from 0.671 to 0.673 remained within a practically equivalent range, indicating that lightweight perturbations in local updates did not alter the global structure associated with behavioral-proxy recovery during the simulation.

Figure 5A shows that the federated configurations preserved analytical utility while maintaining local separation of the original behavioral traces during distributed training, according to the direct-exposure proxy used in this study. All FedAvg scenarios simultaneously maintained high *F*1 values with only moderate communication overhead associated with parameter exchange. The bubble size, proportional to Overlap@10, remained virtually constant across the federated configurations, indicating that explanatory consistency also remained stable under decentralization. This behavior suggests that both the analytical and explanatory structure of the model remained preserved despite local separation of educational behavioral traces across federated clients.

**Figure 5:**
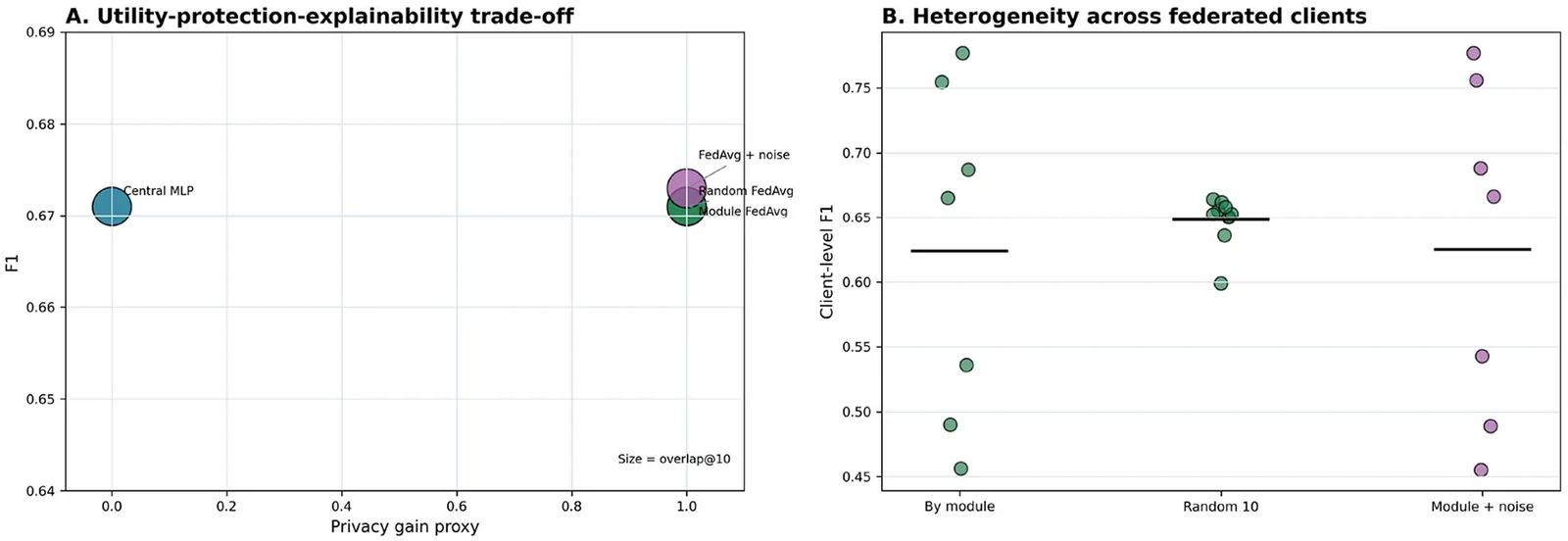
Federated utility, data protection, and heterogeneity between clients. **(A)** Relationship between F1, privacy proxy gain, and explanatory consistency. **(B)** Variation of F1 by client under random partitions by module, with noise.

The distribution illustrated in Figure 5B clearly differentiated the random and academic-module partitioning strategies. Random FedAvg produced a substantially more compact distribution of per-client *F*1 values, with lower overall dispersion and most clients concentrated around the 0.64–0.66 range. In contrast, the FedAvg module configuration exhibited considerably greater variability, with some clients falling below 0.50 while others exceeded 0.75. This behavior demonstrates substantially stronger non-IID heterogeneity when clients preserve educationally interpretable divisions corresponding to actual academic modules.

The FedAvg module + noise configuration preserved a dispersion structure comparable to that observed under the unperturbed academic-module partition, indicating that the principal source of heterogeneity did not originate from the injected perturbations but rather from the uneven distribution of behavioral patterns across educational modules. The coexistence of highly stable clients with considerably less consistent clients additionally revealed that aggregate utility metrics partially masked important local differences in the recoverability of the exploratory proxy.

### 4.5 Explanatory consistency between centralized and federated models

The explanatory structure observed between the centralized MLP and the FedAvg variants remained highly consistent in terms of both dominant-feature composition and relative importance ordering. As presented in Table 6, the centralized MLP, FedAvg module, and FedAvg module + noise configurations preserved the same set of variables within the top-10 explanatory rankings, achieving Overlap@10 = 1.000 across all federated scenarios relative to the equivalent centralized model. Spearman correlation remained similarly high, reaching 0.985 and 0.979, respectively, indicating that decentralization did not substantially reorganize the explanatory hierarchy associated with exploratory behavior.

**Table 6:** Explanatory consistency and dominant behavioral features relative to the centralized MLP.

Model	Top-five features	Overlap@10	Spearman
Central MLP	Temporal dispersion; resource transitions; collaborative interactions; content-resource interactions; assessment-related interaction ratio	1.000	1.000
FedAvg module	Temporal dispersion; resource transitions; content-resource interactions; collaborative interactions; assessment-related interaction ratio	1.000	0.985
FedAvg module + noise	Temporal dispersion; resource transitions; content-resource interactions; assessment-related interaction ratio; collaborative interactions	1.000	0.979

The observed stability was particularly pronounced for temporal and transition-related variables. temporal_dispersion remained the dominant feature across all evaluated configurations, consistently followed by resource_transitions, content_resource_interactions, and collaborative_interactions. This pattern indicates that the explanatory structure of the proxy was primarily dominated by signals associated with temporal distribution of activity, navigation continuity, and transitions among educational resources rather than by isolated measures of interaction intensity.

The variations observed among the federated rankings were limited to minor positional changes among the dominant explanatory variables. In the FedAvg module configuration, content–resource interactions and collaborative interactions exchanged positions relative to the centralized MLP. Likewise, the noisy variant slightly increased the importance of assessment-related interaction ratio. These changes remained small and did not alter the dominant explanatory structure, which continued to be characterized by behavioral dimensions associated with exploration, temporal continuity, and distributed navigation across the VLE.

Figure 6A shows that explanatory similarity between the centralized MLP and the FedAvg variants remained substantially higher than that observed with LR. Ranking correlations between the federated models and the centralized MLP were concentrated near the region of maximum similarity, whereas comparisons involving LR exhibited considerably weaker relationships. This behavior suggests that the observed explanatory differences originated primarily from the model family itself rather than from the federated-training process.

**Figure 6:**
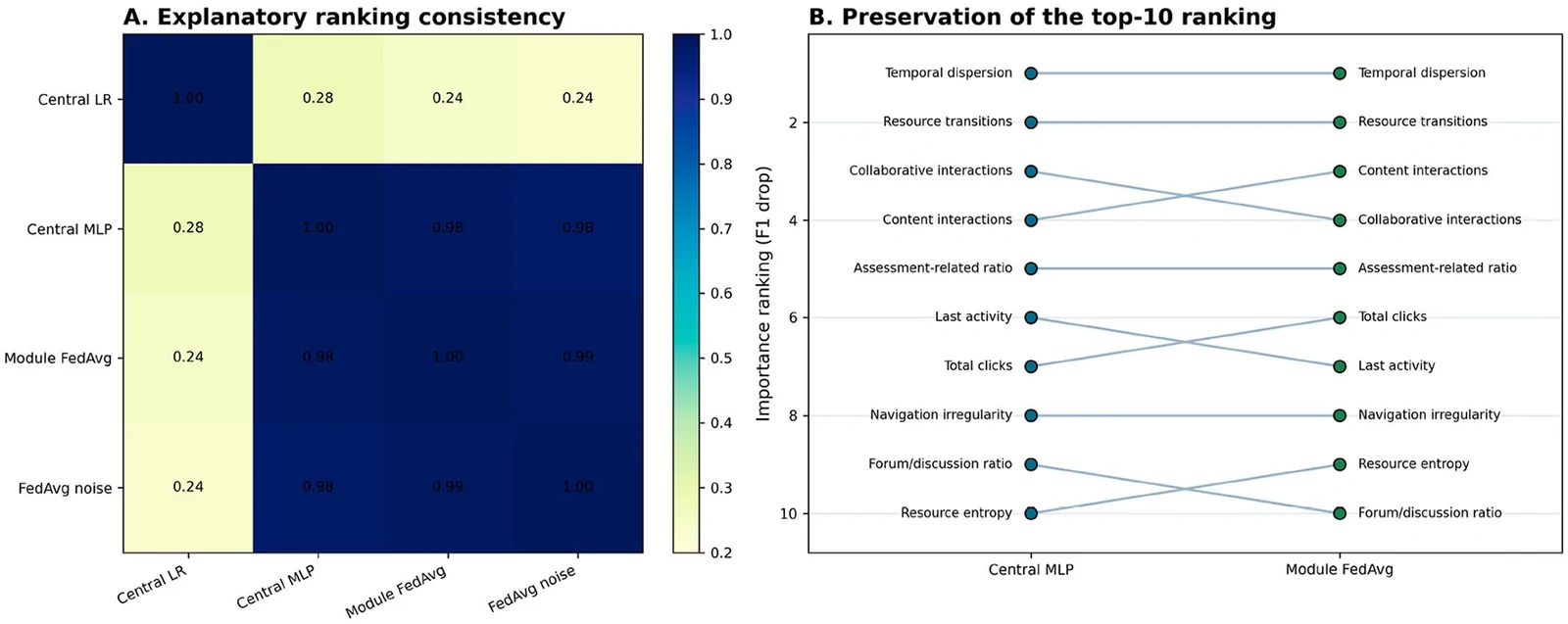
Explanatory consistency between models. **(A)** Correlation of importance rankings between selected models. **(B)** Preservation of the top-10 ranking between central MLP and FedAvg by module.

The preservation of the top-10 explanatory variables illustrated in Figure 6B further demonstrated that decentralization did not eliminate the dominant behavioral signals associated with the exploratory proxy. Variables related to temporal dispersion, resource transitions, and collaborative activity maintained high explanatory importance across both centralized and federated configurations. The structural consistency of the rankings indicates that FL preserved a virtually equivalent explanatory representation of exploratory behavior even under local separation of educational traces across clients.

The observed explanatory stability additionally suggests that redistribution during federated training did not generate excessive dependence on variables associated with specific local subsets of clients. Although academic-module partitioning introduced non-IID heterogeneity into the training process, the dominant explanatory signals preserved a consistent global-importance structure. This behavior became particularly evident through the persistence of temporal_dispersion and resource_transitions as core explanatory dimensions across both centralized and federated configurations.

## 5 Discussion

The results suggest that informal and exploratory behavioral patterns can be operationalized stably from VLE traces without relying on explicit pedagogical labels or traditional academic indicators. The stability observed across the p70, p75, and p80 configurations, together with agreement values close to 0.95 and Jaccard similarity values between approximately 0.80 and 0.83, indicates that the behavioral representation does not depend on an arbitrarily fragile decision boundary but rather on persistent regularities associated with navigation diversity, temporal continuity, and non-evaluative activity. This behavior is consistent with recent studies on exploratory learning and self-directed interaction in educational platforms, where distributed interaction trajectories reflect learning dynamics partially independent of formal evaluative performance (Lin et al., 2024; Vaslin and Prié, 2025). At the same time, the partial stability preserved across the expanded proxy configurations suggests that the exploratory signal remains embedded within the broader interaction structure of the VLE rather than being exclusively tied to a specific operational formulation of the proxy.

Centralized proxy retrieval also produced particularly relevant results. LR achieved *F*1 = 0.977 and AUC-ROC = 1.000 under the complete operational configuration; however, this result must be read as high recoverability of an operationally defined proxy whose components are explicitly trace-based and partly additive. The reduced-control configuration is therefore central to interpretation: it maintained *F*1 = 0.905 and AUC-ROC = 0.991 even after exclusion of the direct proxy components. This behavior suggests that the reconstructed exploratory pattern retains a partially linear and coherent structure within the aggregated behavioral space, while also indicating that the signal is not limited to a direct mechanical reconstruction of the proxy formula.

This finding contrasts with recent literature in educational AI, where increasing architectural complexity is frequently prioritized over interpretability and behavioral traceability (Liu et al., 2026; Yang et al., 2023). In the present study, comparatively lightweight models maintained high retrieval capacity for exploratory behavior without requiring large-scale deep architectures.

The federated configurations produced one of the most relevant findings of the study: FedAvg preserved virtually the same analytical utility as the centralized MLP despite local separation of educational traces and non-IID heterogeneity. Both the centralized scenario and the FedAvg module configuration achieved approximately *F*1 = 0.671 and AUC-ROC = 0.886, while the noisy variant exhibited a marginal improvement in *F*1. This result is particularly relevant because it demonstrates that local separation of raw behavioral traces did not substantially reduce analytical utility under the evaluated configurations. However, the results also showed that global stability coexisted with considerable dispersion among individual clients, especially under academic-module partitioning. This behavior aligns with recent reviews on FL, which indicate that federated systems can preserve aggregate convergence while maintaining substantial structural differences among heterogeneous clients (Khalil et al., 2025; Yurdem et al., 2024). In the present study, heterogeneity manifested not only as statistical variability but also as differences in the recoverability of behavioral patterns associated with specific educational modules.

The explanatory consistency observed between the centralized MLP and FedAvg constitutes another central finding of the study. Permutation importance was used consistently across centralized and federated scenarios because it provides a model-agnostic basis for comparing feature-rank stability under the same behavioral feature space. The results showed Overlap@10 = 1.000 and Spearman correlations close to 0.985 between feature-importance rankings, indicating that decentralization did not substantially alter the dominant interpretive signals. Variables associated with temporal dispersion, transitions between resources, collaborative activity, and interaction with educational content consistently remained among the most relevant explanatory dimensions in both centralized and federated configurations. This result is particularly important because a large portion of the educational federated-learning literature continues to evaluate performance primarily through global predictive metrics without analyzing whether distributed aggregation preserves explanatory coherence between local clients and centralized models. Consequently, the findings suggest that joint evaluation of analytical utility, local trace separation, and interpretive stability can provide a more robust characterization of privacy-aware educational systems than predictive performance metrics alone.

### 5.1 Limitations and future work

The study also presents important limitations. First, the informal/exploratory proxy remains an operational representation derived from behavioral traces rather than a direct pedagogical observation of informal learning. Second, the reduction in direct data exposure achieved through FL should not be interpreted as a formal guarantee of differential privacy, since the framework did not incorporate cryptographic protection, secure aggregation, or formal privacy-preserving mechanisms. Third, federated heterogeneity was simulated through structured partitions within a single educational environment rather than across multiple independent institutions. Fourth, the analysis was intentionally restricted to compact model architectures and model-agnostic explanation methods to preserve reproducibility and interpretability. Consequently, the results should be interpreted as a controlled approximation of non-IID behavior under distributed educational settings. Future work should evaluate the framework in real multi-institutional deployments, incorporate formal differential privacy or secure aggregation, and examine whether dynamic temporal or graph-based representations preserve the same utility–exposure–explainability balance.

## 6 Conclusions

The work presented an integrated framework for analyzing informal and exploratory behavioral patterns in VLE through behavioral learning analytics, FL, and explainability based on behavioral signals. Unlike approaches focused exclusively on academic prediction or predictive performance, the proposed framework aimed to reconstruct interpretable learning trajectories from distributed interaction with VLE resources while simultaneously preserving local separation of educational traces and maintaining explanatory stability under decentralized configurations.

The results suggest that exploratory behavioral patterns can be represented through reproducible proxies derived from navigation diversity, temporal distribution, revisits, and non-evaluative activity. Furthermore, the study demonstrated that analytical utility can be preserved under federated configurations without requiring centralization of raw student traces. More importantly, the stability observed across the explanatory rankings indicates that dominant behavioral signals remained comparatively consistent even after distributed aggregation processes. This behavior is particularly relevant for privacy-aware learning analytics, where preservation of interpretability constitutes an additional challenge beyond maintaining global predictive metrics.

From a methodological perspective, the study additionally showed that comparatively compact models and lightweight federated configurations can capture relevant behavioral regularities without relying on large-scale deep architectures or highly complex analytical pipelines. Consequently, the proposal is positioned primarily as an analytical framework oriented toward interpretable behavioral representation rather than as a system focused exclusively on algorithmic optimization. This orientation enables integration of learning analytics, educational privacy, and XAI within a coherent and reproducible experimental framework.

The work can be extended in several future directions. First, evaluation should be expanded toward real-world multi-institutional scenarios to analyze heterogeneity across independent educational platforms and capture more diverse behavioral distributions. Second, incorporation of formal differential privacy and secure aggregation mechanisms would enable evaluation of the impact of cryptographic constraints on explanatory stability and analytical utility. Finally, future research could explore dynamic representations of exploratory behavior through longer temporal sequences, educational interaction graphs, and adaptive models capable of capturing progressive changes in autonomous learning trajectories within distributed educational ecosystems.

## Data Availability

The datasets analyzed in this study are publicly available from their original repositories. The Open University Learning Analytics Dataset (OULAD) is available at https://doi.org/10.1038/sdata.2017.171, and EdNet is available at https://github.com/riiid/ednet. No new raw data were generated in this study. Additional information regarding data preprocessing, behavioral-layer construction, proxy generation, and analytical configurations is available from the corresponding author upon reasonable request.
